# Zeolitic-Imidazole Framework (ZIF)-Derived ZnO Doped with Ag for Improved Ethanol Sensing Applications

**DOI:** 10.3390/molecules30122611

**Published:** 2025-06-16

**Authors:** Claudio Clemente, Valentina Gargiulo, Luciana Cimino, Giovanni Piero Pepe, Giovanni Ausanio, Ettore Massera, Michela Alfe

**Affiliations:** 1 Institute of Sciences and Technologies for Sustainable Energy and Mobility (CNR-STEMS), 80125 Naples, Italy; claudio.clemente@unina.it (C.C.); valentina.gargiulo@stems.cnr.it (V.G.); luciana.cimino@stems.cnr.it (L.C.); 2 Department of Physics of the University of Naples “Federico II”, 80125 Naples, Italy; giovannipiero.pepe@unina.it (G.P.P.); ausanio@unina.it (G.A.); 3 National Biodiversity Future Center (NBFC), 90133 Palermo, Italy; 4 ENEA Research Center, Portici, 80055 Naples, Italy; ettore.massera@enea.it

**Keywords:** MOF-derived oxides, ZnO, ZIF-8, ZIF-11, ethanol detection, Ag doping

## Abstract

Materials derived from metal–organic frameworks (MOFs) as MOF-derived oxides retain a highly porous and active structure from the MOF precursor, exhibiting excellent sensing properties. In addition, the tunable nature of MOFs allows the structural and chemical properties of the resulting oxides to be specifically tuned to enhance their performance as sensing materials. In this work, zinc-based MOF structures belonging to the family of zeolitic imidazolate frameworks (ZIFs) were synthesized, characterized and then subjected to a high-temperature calcination process to obtain the corresponding oxides. To improve sensing performance, various silver doping strategies (1 wt.%) were explored, specifically through a growth process and an impregnation process. Among these approaches, the oxide obtained via the growth process demonstrates superior performance, exhibiting a response 5.8 times higher than pristine ZnO when exposed to 80 ppm of ethanol at 300 °C in a humidity-controlled chamber. These results highlight the potential of silver doping via growth process as an effective strategy to enhance the sensing performance of MOF-derived ZnO.

## 1. Introduction

Volatile organic compounds (VOCs) and their influence on human health receive a constant attention as personal health concerns develop [1,2,3]. Among VOCs, ethanol is a common hazardous gas that is colorless, volatile and odorous [4]. Exposure to ethanol regularly may cause several health problems, including weariness, headaches, and brain damage [5]. Ethanol gas sensors with high sensitivity, high selectivity, and low detection limit are required in numerous areas, including environmental protection, industrial and food manufacturing, and automobile safety [6]. In recent years, researchers focused on sensors based on metal oxides, such as ZnO [7], Fe_2_O_3_ [8], Co_3_O_4_ [9], because of their remarkable electron properties, variety of detectable gases, low production costs, and adjustable structures [10]. The surface chemistry and microstructure of the sensitive material have a significant impact on the gas-sensitivity reaction process, which may be used to further enhance the performance of gas sensors. As a result, many studies have been published to examine how the surface chemistry of a material affects its sensing performance. These studies include the production and probing of different nanostructures [11,12], the control of exposed facets [12], and the modification of surface defects [13,14]. Furthermore, as sensing materials, researchers have focused emphasis on metal–organic frameworks (MOFs) in the use of gas sensors [15] due to their ultrahigh surface area, porous structure, and customizable and diversified structure [16]. 

MOFs are composed of metal ions and organic ligands which lead to the formation of highly crystalline structures with permanent porosity [17]. Significant research has been carried out on MOFs in the areas of chemical sensors [18], drug delivery [19], catalysis [20], and gas storage and separation [21,22]. To enhance specific functional characteristics (electrical conductivity, sensitivity toward specific chemicals in sensing applications [23]), MOFs can be hybridized and/or mixed with organic or inorganic structures, atoms, or nanoparticles to produce composite materials, or they can be thermally converted into more stable structures. By calcination, MOFs can be transformed into metal oxides with well-designed structures [24].

The permanent porosity of MOFs offers the possibility to confine small metal nanoparticles (MNPs), preventing their migration and aggregation [25]. Among the various synthetic strategies [25,26,27], the surfactant-free introduction of metal precursors into MOFs, followed by in situ reduction to form MNPs@MOF, achieves effective encapsulation with exposed active sites, resulting in high functional performances [26,27,28,29,30]. Following this approach, Zhang et al. [27] developed Ag–ZnO hollow nanocages for the detection of ethanol using ZIF-8 as a sacrificial precursor. They utilized a straightforward method to produce the Ag–ZnO nanocages, which involved the synthesis of a precursor with Ag encapsulated within MOF cavities, followed by a calcination process. The obtained sensors showed higher response and reduced operating temperatures thanks to the catalytic activity of the Ag nanoparticles inside the ZnO structure. Another composite material that showed good sensing properties for ethanol was a hollow hexagonal cylindrical Co-doped In_2_O_3_, obtained by Yong et al. through the calcination of a Co-doped MIL-68(In). The obtained In_2_O_3_, retaining the shape of the MIL-68(In), exhibited a porous, hollow structure with a significant specific surface area. The improved performance shown by the Co-doped In_2_O_3_ was largely attributed to the increased oxygen vacancies introduced by Co doping, which facilitated better gas adsorption and reaction on the sensor surface [31]. Also, it is possible to build various MOF-derived metal oxide nanostructures with effective heterojunction interfaces and porous characteristics for high-performance sensing, starting from bimetallic MOF structures [32]. Li et al. (2022) synthesized nanoporous Co_3_O_4_/TiO_2_ heterojunction nanosheets starting from a mixture of MIL-125 and ZIF-67. The formation of p−n heterojunction at the interface Co_3_O_4_−TiO_2_ enhanced the O_2_ adsorption and thus the sensing properties of the material for ethanol detection [33].

In this work, zinc-based MOF structures belonging to the ZIFs (zeolitic imidazolate frameworks) family were synthesized, structurally characterized, and calcined. Among the extensive range of Zn-based MOFs, ZIF-11 and ZIF-8 are recognized in the literature for their exceptional properties, positioning them as the two most promising ZIFs for gas separation applications [34,35]. ZIF-8 and ZIF-11 were used as sacrificial templates for ZnO production, which was then exposed to a controlled concentration of ethanol in a closed chamber to test the sensing performance at atmospheric pressure and 300 °C. Silver (Ag) doping was employed to enhance the sensing response. Silver was introduced in the ZnO through two doping approaches, starting from MOF or from MOF precursors: (i) MOF impregnation with AgNO_3_ in water suspensions; (ii) solvothermal synthesis from precursors, in which the MOF was allowed to grow from the precursors in the presence of AgNO_3_. The effect of the chemical reduction on hybrid properties was also evaluated. The amounts of AgNO_3_ used in each synthetic protocol were established with the aim of obtaining a final weight load of 1 wt.%. This choice was guided by previous studies [27] which showed that an excessive loading of silver may lead to agglomeration, ultimately reducing the sensing performance. Therefore, a 1 wt.% loading was selected as the optimal balance to enhance material performance.

## 2. Results

### 2.1. ZIF-8 and ZIF-11 and Related Oxides (ZnO(Z8) and ZnO(Z11))

**Figure 1:**
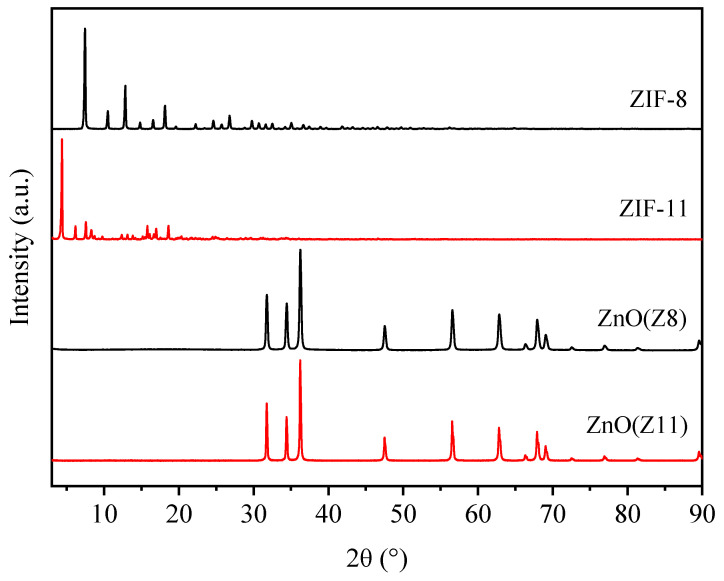
XRD patterns of ZIF-8, ZIF-11, ZnO(Z8), and ZnO(Z11).

An XRD survey was carried out to investigate the crystal structure and phase distribution of ZnO(Z8) and ZnO(Z11) and parent MOFs (ZIF-8 and ZIF-11, respectively). ZIF-8 crystals exhibit strong diffraction peaks in the range of 5° to 50° 2θ, as shown in Figure 1. The main peaks are located at 7.5°, 10.5°, 12.9°, corresponding to the (011), (002), and (112) crystal facets, respectively. The positions and intensities of the diffraction peaks correspond to those reported in the literature for ZIF-8 [34,36,37]. The XRD pattern of ZIF-11 shows prominent diffraction peaks at 4.4°, 6.2°, and 7.6°, corresponding to the (011), (002), and (112) facets. These peaks are consistent with the pattern of ZIF-11 in the rhombic dodecahedron (RHO) type [38,39,40].

The XRD patterns of ZnO(Z8) and ZnO(Z11) exhibit diffraction peaks at 2θ = 31.8°, 34.4°, 36.2°, 47.5°, 56.6°, 62.8°, and 67.9°, corresponding to the (100), (002), (101), (102), (110), (103), and (112) crystal planes of ZnO, respectively, compatible with hexagonal zincite ZnO (PDF Card No. 00-005-0664). The absence of additional peaks indicates that ZnO was obtained without any significant crystal impurity. The average crystallite sizes, estimated using the Scherrer equation, are slightly larger for the ZnO(Z11) sample (61.82 nm) compared to ZnO(Z8) (57.17 nm).

**Figure 2:**
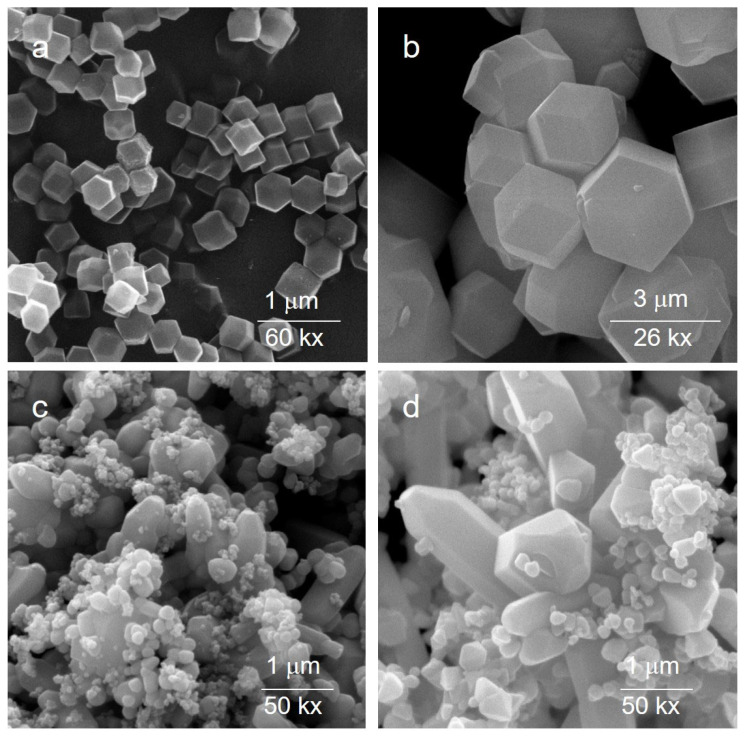
SEM images of ZIF-8 (**a**) and ZIF-11 (**b**) and related oxides (ZnO(Z8) (**c**) and ZnO(Z11) (**d**)).

Figure 2 reports SEM images of the as-prepared ZIF-8 and ZIF-11 crystals. The ZIF-8 crystals have a uniform rhombic dodecahedral shape, with dimensions around 250 nm. They feature smooth surfaces and distinct crystal surface characteristics, consistently with the features reported in the literature [37]. ZIF-11 crystals exhibit a rhombic dodecahedron (RHO) geometry with crystal size range of 1–3 μm.

The morphology of the related ZnO roughly resembles those of the parent MOFs. This is particularly evident in the case of ZnO(Z11), where the RHO geometry remains discernible.

**Figure 3:**
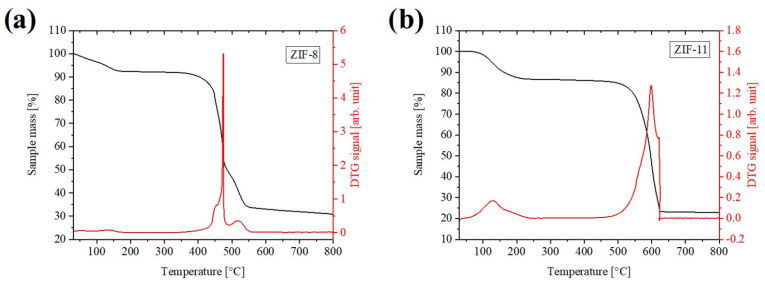
TGA (red) and DTG (black) curves of (**a**) ZIF-8 and (**b**) ZIF-11. Measurements were performed in air at 10 °C/min up to 800 °C.

Figure 3a reports the thermogravimetric (TG) profile of ZIF-8 and ZIF-11 in the 30–800 °C range. ZIF-8 exhibits a lower degradation temperature compared to ZIF-11, which can be attributed to the greater stability of the bIm linker relative to 2-mIm, as demonstrated in previous studies [37]. As concerns the TG profile of ZIF-8, a first weight loss of about 8 wt.% up to 150 °C was observed, which indicated the removal of H_2_O molecules weakly linked in the framework. Above this temperature, ZIF-8 maintains its thermal stability until approximately 350 °C, at which its degradation begins with the maximum degradation rate reached at 470 °C, resulting in a 40% weight loss. A third weight loss of approximately 20 wt.% occurs between 470 °C and 550 °C, attributed to sample inhomogeneity. This additional weight loss is attributed to slight structural variations or differences in crystal size within the sample, leading two distinct degradation steps rather than a single one. The weight stabilizes over 550 °C, yielding 30% of related ZnO.

The ZIF-11 TG profile is reported in Figure 3b and shows an initial weight loss of 12% in the range between 100 and 220 °C, associated with the evolution of the residual toluene entrapped in the pores. Above this temperature, the structure remains stable until 500 °C, after that a noticeable weight loss of 64 wt.% up to 600 °C is detected, associated with the collapse of the ZIF-11 crystals and the formation of the ZnO (around 25 wt.%).

**Figure 4:**
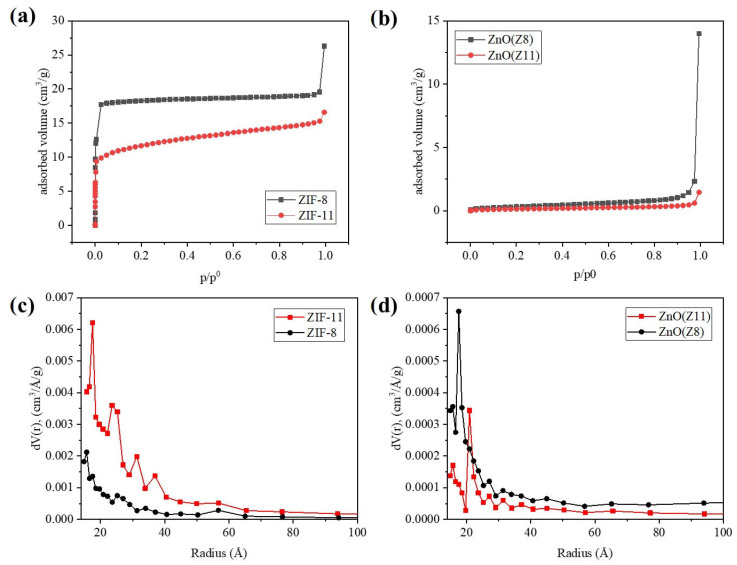
(**a**,**b**) N_2_ adsorption isotherms and (**d**,**c**) the corresponding BJH pore size distribution plots of ZIF-11, ZIF-8 and ZnO(Z11), ZnO(Z8).

The textural properties of the samples were investigated by measuring the N_2_ adsorption isotherms at 77 K. The adsorption curves and the pore size distribution plots are reported in Figure 4. The N_2_ adsorption isotherms of ZIF-11 and ZIF-8 showed a Type I profile, indicating a permanent microporosity (Table 1). The uptake of N_2_ at 77 K of ZIF-11 was lower than that of ZIF-8, probably as a consequence of the different pore diameter (previous reports on ZIF-11 reports 3.0 Å as pore aperture diameter, a value smaller than the kinetic diameter of the nitrogen molecule (3.6 Å) [37]). ZIF-8 was characterized by a surface area of 1843 m^2^/g, while ZIF-11 by 703 m^2^/g. The ZnO samples from ZIF-8 and ZIF-11 were analyzed as well, and they showed a Type IV N_2_ isotherm typical of mesoporous structures. ZnO derived from ZIF-8 exhibited a larger specific surface area (13 m^2^/g) compared to ZnO derived from ZIF-11 (5 m^2^/g), which aligned with the different porosity characteristics of the parent MOF. A difference was detected also in the pore size distributions of the two MOF-derived oxides: ZnO (Z11) was characterized by pores of larger dimensions.

**Figure 5:**
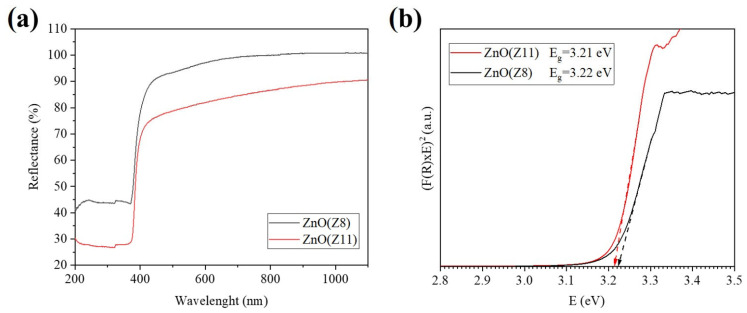
(**a**) Reflectance spectra of pure ZnO from ZIF-11 and ZIF-8; (**b**) Direct bandgap calculation using Kubelka–Munk (K–M) function of ZnO from ZIF-11 and ZIF-8.

UV-visible diffuse reflectance spectroscopy (UV-DRS) is a useful technique for investigating the optical characteristics of semiconductor materials relevant for sensing. The reflectance spectra of ZnO(Z11) and ZnO(Z8) were recorded in the range between 190 and 1100 nm and reported in Figure 5. A blue shift of the reflection peak of ZnO(Z8) compared to ZnO(Z11) is observable. The evaluated Eg values (Eg 3.22 eV (ZnO(Z8)) and 3.21 eV (ZnO(Z11)) are typical of a direct bandgap material [41] and are close to those published in the literature for ZnO [42].

**Figure 6:**
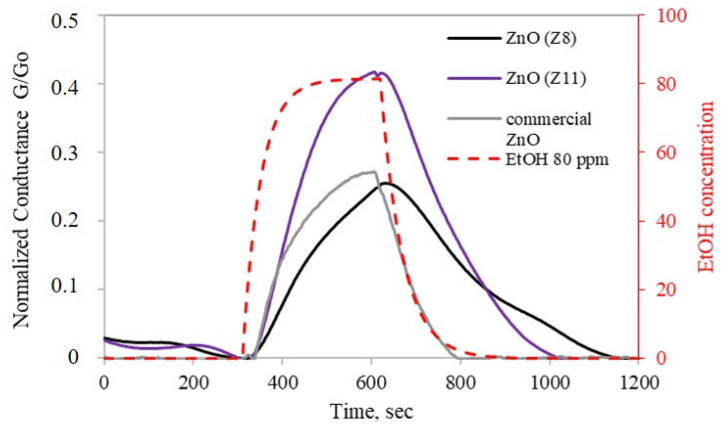
Response of ZnO(Z8) and ZnO(Z11) to 80 ppm ethanol for 10 min as a function of time at 300 °C in dry air. Commercial ZnO has been reported for comparison. The red dashed line represents ethanol concentration.

A preliminary characterization of the sensing properties of ZnO(Z11) and ZnO(Z8) was carried out. For a reliable comparison, the response of commercial ZnO (ReagentPlus^®^, powder, <5 μm particle size, 99.9%, Merck KGaA, Darmstadt, Germany) has been tested under identical experimental conditions. The gold Interdigitated Electrodes (IDEs) coated with ZIF-derived oxides and commercial ZnO were heated, resulting in an increase in conductance until a maximum is reached, which was established as the operative temperature (300 °C). Subsequently, the ZnO layers were exposed to 80 ppm of ethanol and an n-type sensor behavior was observed for both ZnO(Z11) and ZnO(Z8): when the IDE coated with ZIF-derived oxide was exposed to ethanol, an increase in conductance was observed (Figure 6). Although ZnO from ZIF-11 exhibits a lower surface area (Table 1), it showed a higher response towards ethanol (41.7%) than ZnO from ZIF-8 (24.7%) and commercial ZnO. This is possibly due to its slightly lower direct band gap (Figure 5). Moreover, ZnO from ZIF-11 may exhibit fewer structural defects, as suggested by the more defined and ordered structures observed in the SEM images, which can facilitate charge transport and contribute to the enhanced sensing performance.

This survey addressed the selection of ZnO(Z11) to fabricate Ag-doped hybrids (Ag@ZnO(Z11)g, Ag@ZnO(Z11)i,r, and Ag@ZnO(Z11)g,r), with the goal of improving the material response.

### 2.2. Ag@ZnO Derived from ZIF-11

**Figure 7:**
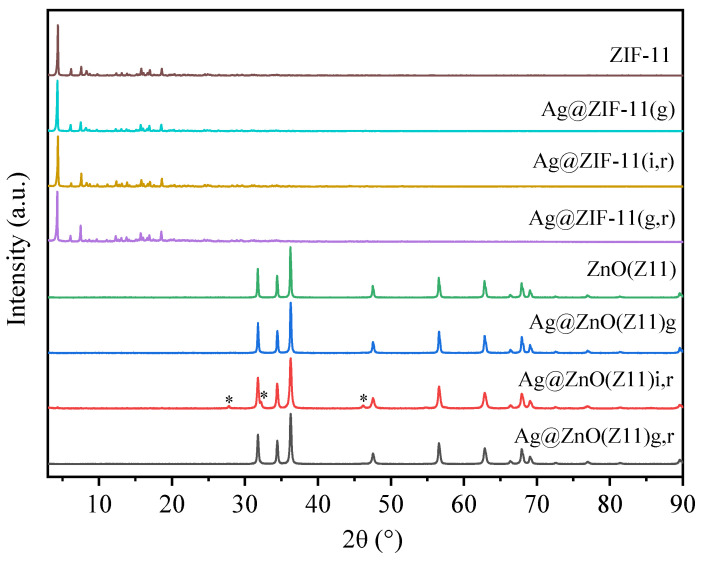
XRD patterns of ZIF-11, Ag@ZIF-11(g), Ag@ZIF-11(i,r), Ag@ZIF-11(g,r) and the corresponding derived-oxides (ZnO(Z11), Ag@ZnO(Z11)g, Ag@ZnO(Z11)i,r, Ag@ZnO(Z11)g,r). The asterisk marks the ZnO phase with a body-centered cubic crystal structure (PDF Card No. 01-079-5604).

Figure 7 reports the XRD patterns of the Ag@ZnO materials derived by ZIF11 according with the approaches reported in Section 3. Additionally, the parent materials are reported to provide a comprehensive understanding of the source and characteristics of the synthesized hybrids.

The Ag@ZIF-11 hybrids exhibit a XRD pattern like the parent ZIF-11, indicating that the incorporation of Ag, even in the case of the MOF obtained by the growth approach (Ag@ZIF-11(g)), does not significantly alter the MOF structure.

The Ag@ZnO samples derived from the calcination of the parent Ag@MOF hybrids show the typical diffraction pattern attributed to hexagonal zincite ZnO (PDF Card No. 00-005-0664). The absence of additional peaks indicates that ZnO was obtained without any significant crystal impurity. Interestingly, the Ag@ZnO(Z11)i sample shows two ZnO crystalline phases: the main hexagonal zincite phase and a minor ZnO phase with a body-centered cubic crystal structure (PDF Card No. 01-079-5604), marked with an asterisk in Figure 7. In contrast, both Ag@ZnO(Z11)g and Ag@ZnO(Z11)g,r samples exhibit only the hexagonal zincite phase. It is noteworthy that in none of the cases are there peaks attributable to elemental Ag; this is due to the superimposition with the ZnO peaks that dominates the diffraction pattern. Moreover, the low silver content in the samples (1 wt.%) may also contribute to the absence of distinct Ag peaks (PDF Card No. 00-004-0783). This observation underscores the effective encapsulation or dispersion of Ag within the ZnO matrix, potentially enhancing the hybrid material’s properties without altering the crystalline structure of the ZnO.

**Figure 8:**
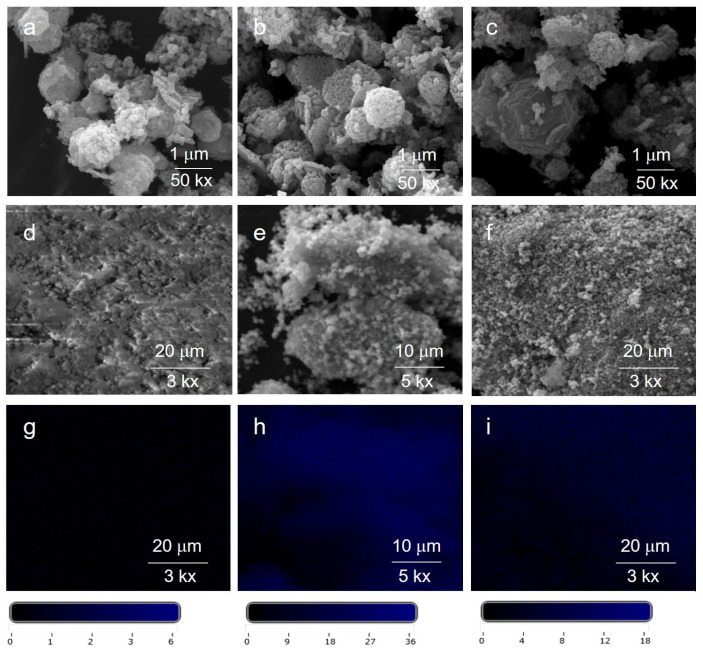
SEM images of (**a**) Ag@ZnO(Z11)g, (**b**) Ag@ZnO(Z11)g,r, and (**c**) Ag@ZnO(Z11)i,r; corresponding lower resolution SEM (**d**–**f**) and EDX Ag elemental mapping (**g**–**i**) with a color scale bar including numerical values that indicate the concentration of Ag in that specific area.

Figure 8 reports SEM images of the Ag@ZnO samples (Ag@ZnO(Z11)g, Ag@ZnO(Z11)g,r, and Ag@ZnO(Z11)i,r). Overall, the rhombic dodecahedron (RHO) geometry of the parent ZIF-11 (Figure 2) remains discernible in all related Ag@ZnO samples. The presence of Ag doping was probed by EDX analysis, which revealed homogeneous dispersion of this element in the sample, as shown in the elemental mapping in Figure 8d–f. The distribution of Ag in the evidenced areas (Figure 8d–f) results higher in the case of the Ag@ZnO(Z11)i,r and Ag@ZnO(Z11)g,r, suggesting that the chemical reduction enhances the Ag density in the samples.

**Figure 9:**
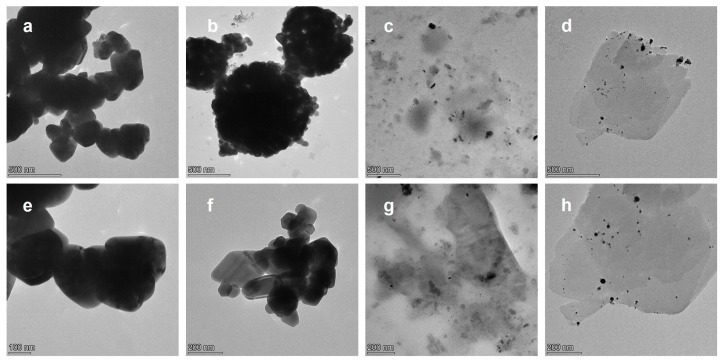
TEM images of (**a**,**e**) ZnO(Z11), (**b**,**f**) Ag@ZnO(Z11)g, (**c**,**g**) Ag@ZnO(Z11)g,r, and (**d**,**h**) Ag@ZnO(Z11)i,r.

Figure 9 presents TEM images of the Ag@ZnO samples (Ag@ZnO(Z11)g, Ag@ZnO(Z11)g,r and Ag@ZnO(Z11)i,r), along with ZnO(Z11) for comparison. Both Ag@ZnO(Z11)g and ZnO(Z11) exhibit a highly homogeneous structure, suggesting—particularly in the case of Ag@ZnO(Z11)g—an efficient incorporation of Ag into the ZnO framework. In the samples Ag@ZnO(Z11)g,r and Ag@ZnO(Z11)i,r, discernible Ag spherical agglomerates with diameters ranging from 5 to 20 nm are observed. Notably, in the case of Ag@ZnO(Z11)i,r (Figure 9d,h), the internal structure of ZnO remains unaltered. Conversely, the internal structure of Ag@ZnO(Z11)g,r (Figure 9c,g) exhibits inhomogeneities, which can be attributed to the presence of reduced silver within the material.

**Figure 10:**
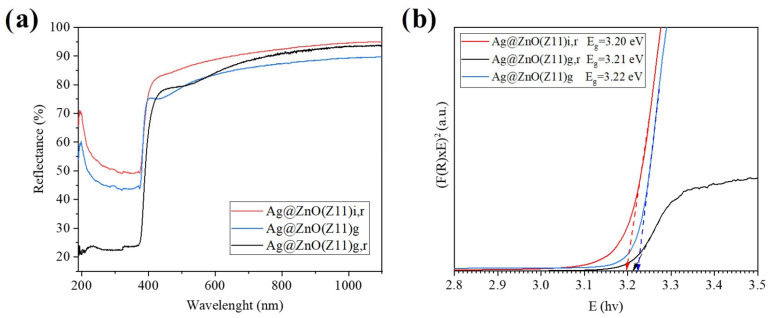
(**a**) Reflectance spectra of Ag-doped ZnO samples; (**b**) Direct bandgap calculation using Kubelka–Munk (K–M) function of Ag-doped ZnO samples.

Figure 10 reports the reflectance spectra of the Ag@ZnO samples (Ag@ZnO(Z11)g, Ag@ZnO(Z11)g,r and Ag@ZnO(Z11)i,r,) together with the direct bandgap calculation using the Kubelka–Munk (K–M) function (Section 3). It is interesting to evidence a slight increase in the apparent bandgap energy of Ag@ZnO(Z11)g,r and Ag@ZnO(Z11)g (3.21 and 3.22 eV, respectively, Figure 10b) compared to Ag@ZnO(Z11)i,r (3.20 eV). This slight increase in bandgap energy is attributed to the Moss–Burstein effect, which occurs due to the accumulation of additional electrons in the conduction band because of oxygen vacancies. The presence of these vacancies leads to a higher electron concentration, causing the Fermi level to shift into the conduction band and thereby increasing the observed bandgap energy resulting in a broader bandgap in zinc oxide [43]. The differences in electronegativity and ionic radius between silver and zinc, combined with the partial substitution of silver into the zinc lattice, lead to the formation of oxygen vacancies and an increase in electron density. These modifications enhance the electronic properties of the material, contributing to its improved sensing performance [44].

**Figure 11:**
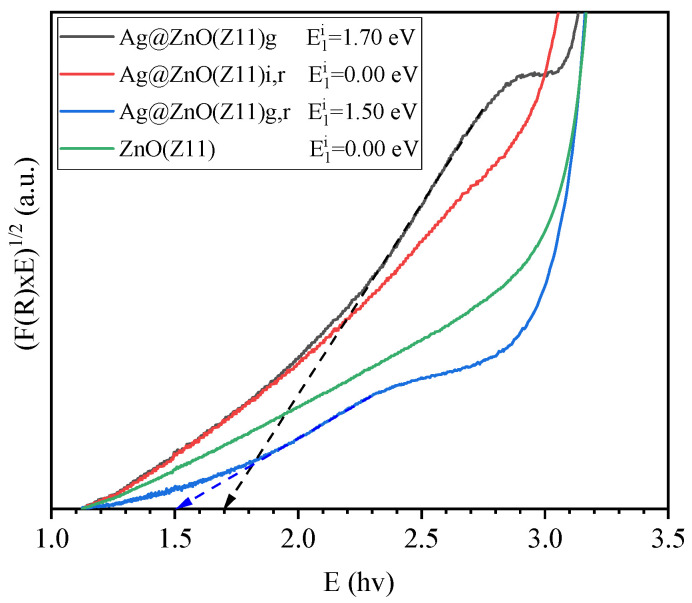
Plots of indirect optical transitions calculation in the range of 1–3.5 eV using Kubelka–Munk (K–M) function of ZnO(Z11) and Ag-doped ZnO(Z11) samples.

In addition to direct bandgap transitions, ZnO excitation can occur also via indirect transitions caused by defect formation and/or doping [45]. In the [F(R) × hv]1/2 plot (Figure 11), indirect bandgap transitions are analyzed. This approach allows for the detailed examination of the optical properties influenced by the defects or dopants, providing insights into the electronic structure and behavior of the material examined. For Ag@ZnO(Z11)g and Ag@ZnO(Z11)g,r samples, indirect transitions are observed in the energy range 2.0–3.0 eV, which are not observed in the other samples. Plotting the regression line in this interval, a bandgap value of 1.70 and 1.50 eV for Ag@ZnO(Z11)g and Ag@ZnO(Z11)g,r, respectively, are calculated. These emerging energy levels can be attributed to the formation of a new acceptor level. Consequently, the observed shoulder is associated with the excitation from the valence band to this acceptor level. Boulahlib et al. observed the same effect in ZnO doped with Ag and demonstrated that it is dependent on Ag-content [46].

**Figure 12:**
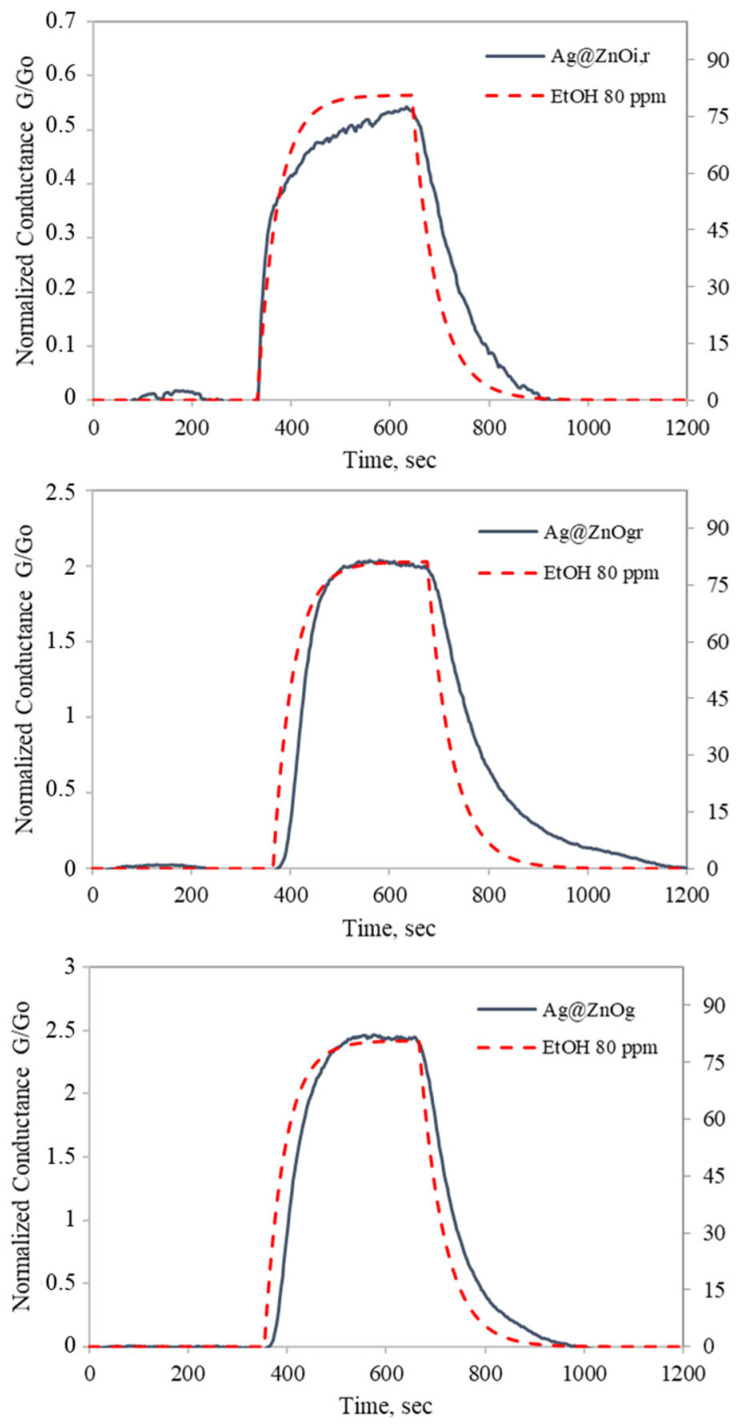
Response of ZnO(Z11), Ag@ZnO(Z11)i,r, Ag@ZnO(Z11)g, Ag@ZnO(Z11)g,r to 80 ppm ethanol for 10 min as a function of time at 300 °C in dry air. The red dashed line represents ethanol concentration.

In Figure 12, the responses of Ag@ZnO samples to 80 ppm of ethanol are reported. The Ag-doped ZnO samples exhibit a greater current drop upon activation to their operational temperature, attributed to the catalytic effect of silver on oxygen molecules. As a result, more oxygen was adsorbed on the surface, leading to an increased number of active sites that reacted with ethanol gas, thereby enhancing the material’s response. The percentage-normalized conductance responses of Ag@ZnO(Z11)i,r, Ag@ZnO(Z11)g, and Ag@ZnO(Z11)g,r are 51%, 243%, and 202%, respectively. Among these, Ag@ZnO(Z11)g exhibits the highest response and the greater baseline stability. This is evident from Figure 12, where the conductance returns to its baseline after ethanol exposure. This behavior indicates the effective catalytic activity of Ag@ZnO(Z11)g, facilitating a rapid recovery and enhancing the sensing performance. The improved performance can be attributed to the formation of a new acceptor level as a result of silver doping, as indicated by the optical analysis (Figure 10 and Figure 11).

The adsorption-reaction mechanism is the more probable for oxide-based gas sensors exposed to reducing gases [47,48,49]. The adsorption-reaction mechanism can be described by the following equations:

O_2_(ads) + 2e^−^ → 2O^−^(ads) (1)

CH_3_CH_2_OH(ads) + 6O^−^(ads) → 2CO_2_(ads) + 3H_2_O(ads) + 6e^−^ (2)

The ambient oxygen molecules are adsorbed on the surface of ZnO, trapping free electrons from its interiors and oxygen ions (O^−^) are formed [Equation (1)]. As a result, a thick layer of electron depletion forms at the ZnO surface, decreasing the conductance of the sensor [50]. When ethanol flows in, the adsorbed O^−^ ions react with ethanol molecules and electrons are released [Equation (2)], reducing the thickness of the space-charge layer and potential barrier [51]. This allows the conductance to increase and improves the gas sensing response. Catalytic sensitization effects of Ag doping also help to improve ethanol sensing performances [52]. Ag particles offer additional active sites for the dissociation of a larger number of adsorbed oxygen molecules [O_2_(ads)], resulting in more surface-absorbed O^−^ ions reacting with ethanol [53]. Improving the response to detect ethanol, the doping of ZnO with Ag can allow to reduce the optimal working temperature of the sensor [27].

## 3. Materials and Methods

### 3.1. Materials

Benzimidazole (bIm, C_7_H_6_N_2_, 98 wt.%), methanol (CH_3_OH 99.8 wt.%), toluene (C_6_H_5_CH_3_, 99.8 wt.%), ammonium hydroxide aqueous solution (NH_4_OH, 28–30 wt.% NH_3_ basis), zinc acetate dihydrate (Zn(CH_3_COO)_2_⋅2H_2_O, ≥98 wt.%), 2-methylimidazole (2-MeIm, C_4_H_6_N_2_, 99 wt.%), acid L-ascorbic (C_6_H_8_O_6_), silver nitrate (AgNO_3_), and barium sulfate (BaSO_4_) were purchased from Merck KGaA, Darmstadt, Germany and used as received. The gold interdigitated electrodes (IDEs) used for the survey were commercially available featuring gold electrodes on a ceramic substrate (Metrohm DropSens, Oviedo, Spain, IDEAU200-HPT-WB). The dimensions for bands/gaps were 200 μm and ceramic substrate dimensions were 22.8 mm × 7.6 mm × 1 mm.

### 3.2. Synthesis of ZIF-8

ZIF-8 was synthesized using a typical hydrothermal approach with water as solvent. In a typical synthesis, 0.4085 g of Zn(Ac)_2_∙2H_2_O were dissolved in 5 mL of deionized (DI) water and 2.2919 g of 2-MeIm were dissolved in 15 mL of DI water. The two clear solutions were mixed and stirred to obtain a final solution with a molar ratio of Zn^2+^: 2-MeIm = 1:15 and then transferred into a 50 mL Teflon-lined autoclave and heated at 140 °C for 24 h. After cooling down the system, ZIF-8 crystals were recovered by under-vacuum filtration and purified by several washings with acetone. Subsequently, the product was dried at 70 °C overnight. The ZIF-8 yield was 93.6%, based on the metal content.

### 3.3. Synthesis of ZIF-11

In a typical process, 0.5510 g of Zn(Ac)_2_∙2H_2_O were dissolved in 15 mL of methanol and 0.6019 g of bIm were dissolved in 15 mL of methanol. After complete dissolution, 27 mL of toluene and 0.2 mL of ammonium hydroxide were also added. The metal-containing solution was added dropwise into the organic linker solution at a rate of ~60 drops/min under magnetic stirring. The Zn: bIm molar ratio in the final synthetic mixture was 1:2. The mixture was stirred at room temperature for 4 h. The ZIF-11 crystals were recovered by under-vacuum filtration and purified by several washings with methanol. Subsequently, the product was dried at 50 °C overnight. The ZIF-11 yield was 92.9%, determined based on the metal content.

### 3.4. Synthesis of Ag@ZIF-11 Hybrids

**Figure 13:**
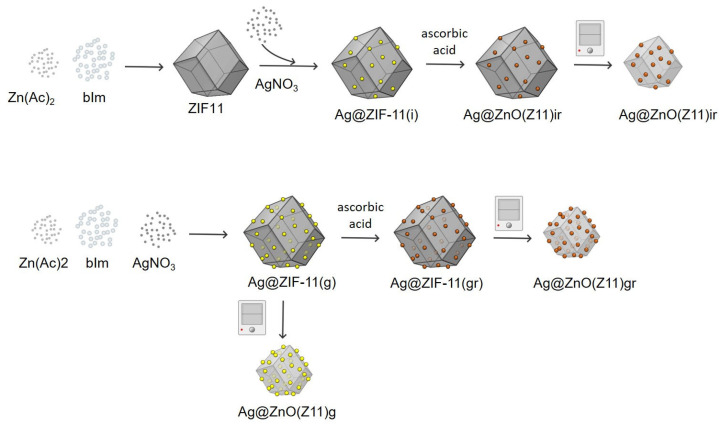
Scheme of the synthetic strategies adopted to produce Ag@ZIF-11 hybrids and resulting oxides.

Ag@ZIF-11 hybrids were synthesized by applying two approaches (Figure 13): (i) impregnation method (sample labeled as Ag@ZIF-11(i)) in which the ZIF-11 structure was impregnated with AgNO_3_ in water solution; (ii) growth process, in which the ZIF-11 was allowed to grow in the presence of AgNO_3_ (sample labeled as Ag@ZIF-11(g)). To evaluate also the effect of the chemical reduction on the hybrid properties, part of the obtained materials was chemically reduced (reduced samples were also labeled with “r” where appropriate). The amounts of AgNO_3_ used in each synthetic protocol were established with the aim of obtaining a final weight load of 1 wt.%.

Ag@ZIF-11(i) synthesis: A total of 100 mg of ZIF-11 was dispersed in 10 mL of methanol, and 10 mg of AgNO_3_ was dissolved in 10 mL of methanol. Then, 1.6 mL of AgNO_3_ in methanol solution was introduced into the ZIF-11 suspension (corresponding to the volume containing the amount of Ag ions necessary to obtain a final theoretical load of 1 wt.%). The resulting mixture was stirred for 2 h and then the product was recovered by under-vacuum filtration and labeled as Ag@ZIF-11(i).

Ag@ZIF-11(g) synthesis: During the ZIF-11 synthesis, 0.0110 g of AgNO_3_ were added to the Zn(Ac)_2_ solution and the resulting solution was introduced dropwise into the organic linker, one at a rate of ~60 drops/min under magnetic stirring. Then, the reaction conditions were kept as those described before for the synthesis of ZIF-11. The work-up of the mixture at the end of the reaction was the same as described before. The obtained sample was labeled as Ag@ZIF-11(g).

Chemical reduction: A total of 100 mg of Ag@ZIF11(i) or Ag@ZIF11(g) was dissolved in methanol and treated with 20 mg of ascorbic acid for 2 h at room temperature under stirring. Then, the product was recovered by under-vacuum filtration, washed with methanol several times and then dried at 50 °C. The obtained samples were labeled as Ag@ZIF-11(g,r) and Ag@ZIF-11(i,r).

### 3.5. Synthesis of ZnO and Ag@ZnO

ZnO samples from ZIF-11 and ZIF-8 and Ag@ZnO samples from Ag@ZIF hybrids were obtained by calcinating each precursor powder at 650 °C for 1.5 h in air in a muffle. The samples obtained were named according to Table 2.

### 3.6. Characterization

X-ray powder diffraction (XRD) analysis was performed on powder samples using a Rigaku MiniFlex 600 diffractometer (Tokyo, Japan) in the 2θ range 3–90°. Crystal phase attribution was carried out using the PDF-5 2024 (International Center for Diffraction Data, Tokyo, Japan) database and Rigaku PDXL2 (Rigaku, Tokyo, Japan) software SmartLab Studio II (SLSII).

A FEI Inspect™ S50 Scanning Electron Microscope (SEM) (Thermo Fisher Scientific Inc., Waltham, MA, USA) was used to investigate the morphology of the materials. The powdered samples were previously dried, then underwent gold sputter-coating to prevent local charging.

TEM images were acquired on a Thermofischer Talos L120C G2, LaB6 source, 120 kV, equipped with a Ceta 16M Camera. The powdered samples were suspended in water and deposited onto carbon film-supported copper grid, standard thickness, grid size 400 mesh.

Thermogravimetric analyses were performed on a PerkinElmer STA6000 thermogravimetric analyzer (PerkinElmer, Inc., Waltham, MA, USA) (gas flow 40 mL min^−1^) in oxidative (air) conditions, from 30 °C to 800 °C at a heating rate of 10 °C min^−1^. To accurately evaluate mass losses, samples were put into an alumina crucible that had been thermally preconditioned to 950 °C.

Textural characteristics were determined by N_2_ adsorption at 77 K on a Quantachrome Autosorb 1. The samples were outgassed under vacuum at 120 °C for 3 h before the analysis, and the results were processed using the BET equation for SA assessment and the BJH model for pore size distribution evaluation.

UV-visible diffuse reflectance spectroscopy (UV-DRS) was performed on an ISR-2600 plus Integrating Sphere-supported UV-vis-NIR spectrophotometer (2600 Series, Shimadzu, Japan). The calibration was performed using barium sulfate powder, which yields 100% reflectance. To prevent instrumental interference, the same barium sulfate powder was used for the baseline measurement.

The Kubelka–Munk (K–M) function was used for calculating the optical bandgap energy (E_g_) from the UV-DRS spectra [54]. The Kubelka–Munk remission function, F(R), is based on the equation below.

(3) $$F\left(R\right)=\frac{{\left(1-R\right)}^{2}}{2R}$$

where R represents reflectance; F(R) is related to the absorption constant of the material [55].

(4) $${\left(F\left(R\right)hv\right)}^{1/n}=B(hv-E_{g})$$

where h is the Planck constant, ν is the frequency of the photon, E_g_ is the bandgap energy, and B is a constant. The n factor is determined by the kind of electron transition and is equal to 1/2 or 2 for direct and indirect transition band gaps evaluation, respectively [36]. To estimate the bandgap, [F(R) × hv]^1/n^ against hv was plotted and the E_g_ value was evaluated by extrapolating the linear part of the graph to the energy axis at [F(R) × hυ]^2^ = 0.

The average crystallite size was calculated using the Scherrer equation:

(5) $$D=\frac{K\lambda }{\beta cos\theta }$$

where D is the crystallite size (nm), K is the shape factor (0.94), λ is the X-ray wavelength (Cu-Kα, λ = 0.1541 nm), β is the full width at half maximum (FWHM) of the peaks and θ is the Bragg angle.

### 3.7. Ethanol Sensing Performances Evaluation

The obtained ZnO samples were gently pressed onto the IDE surface. To improve the adhesion to the substrate, the system underwent a thermal treatment at 400 °C for 1 h in a muffle furnace under air. The thickness of the sensing layers was 8 ± 1 μm (the thickness evaluation was obtained with a Bruker DektakXT Stylus Profiler), and the sensing layer was 5 × 5 mm^2^. The gas sensing tests were performed in dry conditions at atmospheric pressure and at 300 °C in a sealed chamber. For gas-sensing characterizations, the ethanol was stored in approved cylinders as a calibrated vapor balanced in dry air at 100 ppm (Ossi Gas SRL). Computer mass flow controllers (MKS) and electro-pneumatic valves were utilized to accurately and quickly adjust the gas flow mixing rates. A bias voltage of 1 V was provided to the electrodes using a Precision Power Supply TTi QL355T, and conductance values were measured using a high-resolution Keithley 6485 Picoammeter. A steady flow of 500 sccm of synthetic dry air was used as a gas carrier and combined with the necessary concentration of ethanol. The IDEs were exposed to the ethanol for 10 min after 30 min in dry air (baseline). To compare different devices, a response is defined as the normalized relative variation in conductance $\left|{∆G}_{10}-G_{0}\right|/G_{0}$, where G_0_ is the base conductance measured before exposure to the analyte, and G_10_ is the conductance after 10 min of exposure.

## 4. Conclusions

ZIF-11-derived ZnO doped with Ag (weight load 1 wt.%) have been obtained by different approaches (impregnation/reduction, growth process, growth process/reduction) and tested for ethanol detection (80 ppm, humidity-controlled chamber, atmospheric pressure, working temperature 300 °C). The doping with Ag ions results in band structure changes measured as variation in the apparent band gap and in the emerging energy levels due to indirect transitions, with extrapolated values of 1.70 eV and 1.50 eV for Ag@ZnO(Z11)g and Ag@ZnO(Z11)g,r, respectively. The enhanced gas sensing performance is attributed to the increased number of active sites, facilitating the dissociation of adsorbed oxygen molecules into O⁻ ions that react with ethanol. Hence, this research highlights the potential of MOF-derived oxides doped with silver nanoparticles for gas sensing applications. It also shows the potential of using less common MOFs, like ZIF-11, as active precursors, encouraging the exploration of alternatives beyond the well-known ZIF-8. Moreover, the study demonstrates that doping during MOF synthesis in a one-pot approach, rather than as a post-synthesis step, can offer clear advantages, opens new possibilities for designing efficient and high-performance sensing materials.

## Figures and Tables

**Table 1:** Specific surface area (SSA) and total volume of the adsorbent materials.

Material	Specific Surface Area (m^2^/g)	Total Volume (STP) cm^3^/g
ZIF-8	1843	0.969
ZIF-11	703	0.418
ZnO(Z8)	13	0.213
ZnO(Z11)	5	0.020

**Table 2:** Samples labels.

Sample Name	Parent Material	Synthetic Approach
Ag@ZIF-11(i)	ZIF-11	Impregnation
Ag@ZIF-11(g)	-	Solvothermal from precursors
Ag@ZIF-11(i,r)	Ag@ZIF-11(i)	(i) Impregnation; (ii) Chemical reduction
Ag@ZIF-11(g,r)	Ag@ZIF-11(g)	(i) Solvothermal from precursors; (ii) Chemical reduction
ZnO(Z8)	ZIF-8	Calcination
ZnO(Z11)	ZIF-11	Calcination
Ag@ZnO(Z11)i	Ag@ZIF-11(i)	Calcination
Ag@ZnO(Z11)g	Ag@ZIF-11(g)	Calcination
Ag@ZnO(Z11)i,r	Ag@ZIF-11(i,r)	Calcination
Ag@ZnO(Z11)g,r	Ag@ZIF-11(g,r)	Calcination

## Data Availability

Dataset available on request from the authors.
